# New water-based nanocapsules of poly(diallyldimethylammonium tetrafluoroborate)/ionic liquid for CO_2_ capture

**DOI:** 10.1016/j.heliyon.2023.e13298

**Published:** 2023-01-30

**Authors:** Bárbara B. Polesso, Rafael Duczinski, Franciele L. Bernard, Douglas J. Faria, Leonardo M. dos Santos, Sandra Einloft

**Affiliations:** a Post-Graduation Program in Materials Engineering and Technology, Pontifical Catholic University of Rio Grande do Sul – PUCRS, Brazil; b School of Technology, Pontifical Catholic University of Rio Grande do Sul – PUCRS, Brazil

**Keywords:** Green chemistry, poly(ionic liquid) nanocapsules, Ionic liquids, Encapsulation, CO_2_ capture

## Abstract

Encapsulated ionic liquids as green solvents for CO_2_ capture are reported in this work. We present a novel combination of water-based poly(ionic liquid) and imidazolium-based ionic liquids (Emim[X]). Poly(diallyldimethylammonium tetrafluoroborate)/Emim[X] capsules were developed for the first time using Nano Spray Dryer B-90. Capsules were characterized by FTIR, SEM/EDX, TEM, TGA, DSC, CO_2_ sorption, and CO_2_/N_2_ selectivity, CO_2_ sorption kinetic and recycling were also demonstrated. Comparing the capsules reported in this work, the combination of poly(diallyldimethylammonium tetrafluoroborate) and the ionic liquid 1-ethyl-3-methylimidazolium tetrafluoroborate (P[DADMA]/BF_4_) showed great potential for CO_2_ capture and CO_2_/N_2_ separation, providing higher results (53.4 mg CO_2_/g; CO_2_/N_2_ selectivity: 4.58).

## 1 Introduction

Combustion of fossil fuel and industrial processes are largely responsible for anthropogenic CO_2_ emissions in the atmosphere. CO_2_ capture and storage technologies are suggested as the easiest and most effective way to reduce CO_2_ emissions at a large scale [[1], [2], [3]]. Current amine-based CO_2_ capture systems, have some drawbacks such as high regeneration energy, equipment investment and corrosion [4].

The search for novel materials for CO_2_ capture combining efficiency and safety, besides being sustainable processes is urgent. Despite water being considered safe, non-toxic, and environmentally friendly, it is not always compatible with the process systems. Green solvents are emerging as benign when compared to conventional solvents toxicity [5,6]. Ionic liquids (ILs) are classified as green solvents due to their low vapor pressure, non-flammability, and recyclability. Other properties such as tunability and high thermal stability also draw attention to this material [7,8]. Simulation studies showed that the energy consumption of the IL-based process can be 26% lower than the amine-based process [9]. However, high viscosity and low CO_2_ sorption rate represent a challenge to its use [[10], [11], [12]]. Solvent encapsulation combines the advantages of liquid solvents and solid sorbents, overcoming the mass transfer limitation. Encapsulated ionic liquid has been proposed as a potential strategy for CO_2_ capture, increasing the contact between the gas and liquid phases [[13], [14], [15], [16]]. Encapsulated ionic liquid improved CO_2_ sorption rocess, showing higher mass transfer rates when compared with neat ionic liquids [14,[17], [18], [19]]. Recently works present the combination of polymer shell (polysulfone [19,20], silicone [18,21,22], acrilates [22]) and ionic liquids using different encapsulation techniques as emulsification [19,20,23], UV [10,22], polymerization [23] and impregnation [13,14]Studies also indicated that encapsulated ionic liquid can be successfully regenerated under mild conditions and used in consecutive CO_2_ sorption/desorption cycles, without operation efficiency loss [13,16,18,21]. Knipe el al. presented ionic liquid encapsulation as a potential substitute for amines, since silicone capsules of [P_2222_][BnIm] and [P_2228_][2CNPyr] perfomed similar or even better for CO_2_ sorption than aqueous amines at low pressure and 25 °C [21].

Process criteria such as thermal stability, solvent regeneration, compatibility, and CO_2_ permeability can be affected by shell material choice [22]. Poly(ionic liquid)s or polymerized ionic liquids (PILs) combine polymers (processability, film-forming properties, etc) and ILs proprieties (high termal stability, affinity to CO_2_, etc) emerging as high permeable membranes for CO_2_ separation. Studies suggest that PILs are very selective for CO_2_ separation from CO_2_/N_2_ mixture, showing better results for CO_2_ separation when compared with ILs. CO_2_ sorption capacity can be strongly affected by PIL cation, presenting better results with ammonium-based ones [[24], [25], [26], [27]]. The use of water-based PILs to encapsulate IL contributes to the advance of scientific knowledge and technology for CO_2_ capture, but also is in agreement with the green chemistry principles. It must be emphasized that the use of nanospray dryer allied to water as solvent in the encapsulation process offers a new platform to environmental benign syntheses, the use of alternative solvents and atom economy since the yield of the nanospray is higher compared to conventional spray dryer processes [28,29]. As far as we know, capsules combining water-based PILs as shell and ILs as core are a new approach for CO_2_ capture.

Herein, we report the IL 1-Ethyl-3-methylimidazolium with different anions (Emim[X]) encapsulation, using as shell the water-based PIL poly(diallyldimethylammonium tetrafluoroborate). The encapsulation process using Nano Spray Dryer B-90 and water as solvent was also described. Yet, CO_2_ sorption and CO_2_/N_2_ selectivity, process parameters, thermal stability and recyclability were also evaluated.

## 2 Experimental

### 2.1 Materials

Aqueous solution of poly(diallyldimethylammonium chloride), P[DADMA][Cl] (20 wt%, mw. 400,000–500,000), Lithium tetrafluoroborate salt (98%), 1-Ethyl-3-methylimidazolium methanesulfonate, Emim[MSO_3_] (95%), 1-Ethyl-3-methylimidazolium trifluoromethanesulfonate, Emim[CF_3_SO_3_] (98%), were purchased from Merck. The chemicals were used without further purification. CO_2_ (99.8%) and CO_2_/N_2_ (15.94%/balance) were purchased from White Martins.

First, the Poly(ionic liquid) poly(diallyldimethylammonium tetrafluoroborate) (P[DADMA][BF_4_]), used as shell material, was obtained by anion exchange from Li[BF_4_] and an aqueous solution of poly(diallyldimethylammonium chloride), P[DADMA][Cl], following literature procedures [30]. The Ionic liquids 1-Ethyl-3-methylimidazolium bromide (Emim[Br]) and 1-Ethyl-3-methylimidazolium tetrafluoroborate (Emim[BF_4_]), used as core, were synthesized as described in literature [31,32]. Proton Nuclear Magnetic Resonance (1H-NMR) (Varian spectrophotometer, VNMRS 300 MHz). Emim[Br]: H NMR δ 1.57 (t, 3H), 4.11 (s, 3H), 4.42 (q, 2H), 7.56 (s, 2H), 10.19 (s, 1H) Emim[BF_4_]: 1H NMR 1.43 (t, 3H), 3.84 (s, 3H), 4.15 (q, 2H), 7.37 (s, 1H), 7.43 (s, 1H), 8.63 (s, 1H).

### 2.2 Ionic liquid encapsulation

**Fig. 1:**
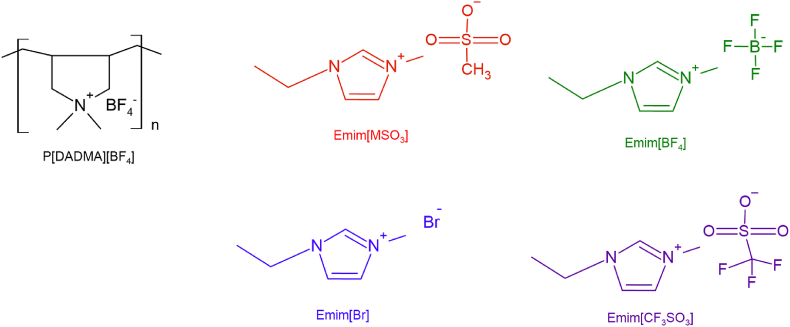
Structure materials used to ENILs.

Ionic liquid Emim[X] was encapsulated in P[DADMA][BF_4_] by spray drying using a Nano Spray Dryer B-90 (BÜCHI Labortechnik AG, Flawil, Switzerland) equipment. The materials structure of encapsulated ionic liquids (ENILs) can be observed in Fig. 1.

For the encapsulation process, P[DADMA][BF_4_] was dissolved in distilled water under mild heating. After complete dissolution, the ionic liquid was added and the solution was stirred until the complete homogenization. The solution was fed to the atomization system using a peristaltic pump. Mass proportion of P[DADMA][BF_4_]:Emim[X] was 1:0.5. Nano Spray Dryer process parameters (Table 1) were previously tested and defined using distilled water in the equipment.

**Table 1:** Nano Spray Dryer process parameters.

Parameters	
Frequency (kHZ)	110
Spray (%)	80
Pump (%)	36–46
Inlet Temperature (°C)	107
Spray mesh size	Small

Yield was obtained using mass balance and encapsulation efficiency (EE) was calculated by equation (1) [33].

(1) $$EE\;\left(\%\right)=\frac{w_{IL-s}}{\left(\frac{w_{IL-e}}{Y}\right)}\times 100$$

Where w_IL-s_: IL mass fraction obtained by acetone extraction method [20]; w_IL-e_: IL theoretical mass fraction; Y: Yield.

Aiming to compare results for both configurations – capsules and the pristine PIL (P[DADMA]-poly) – capsules were obtained (without IL - P[DADMA]-cap). Sample code and its components are shown in Table 2.

**Table 2:** Sample code.

Sample Code	Components
C	P[DADMA][BF_4_] capsules
P[DADMA]-poly	P[DADMA][BF_4_] polymer
P[DADMA]/MSO_3_	P[DADMA][BF_4_]/Emim[MSO_3_] capsules
P[DADMA]/BF_4_	P[DADMA][BF_4_]/Emim[BF_4_] capsules
P[DADMA]/Br	P[DADMA][BF_4_]/Emim[Br] capsules
P[DADMA]/CF_3_SO_3_	P[DADMA][BF_4_]/Emim[CF_3_SO_3_] capsules

### 2.3 Encapsulated ionic liquids characterization

Scanning electron microscopy with field emission (SEM-FEG) using FEI Inspect F50 in the secondary electron mode (SE) was performed to evaluate particle morphology and size. Chemical composition was assessed by energy dispersion X-ray spectrometry (EDX). FTIR spectra were recorded on a PerkinElmer Spectrum100 spectrometer in UATR mode. Particle structure was assessed by transmission electron microscopy (TEM) (Model Tecnai G2 T20 FEI). Thermal stability was investigated by TGA (TA Instruments SDT-Q600), under nitrogen atmosphere with a temperature range from 25 to 700 °C and a heating rate of 20 °C/min. Differential scanning calorimetry - DSC (TA Instrument Q20) was performed from −90 °C to 100 °C at a heating rate of 5 °C/min under nitrogen atmosphere. IL encapsulated amount (% IL) was measured by the acetone extraction method (performed in triplicates) [20]. CO_2_ sorption tests were performed using the well-known pressure decay technique [34,35]. CO_2_/N_2_ selectivity was performed using the same method coupled to gas chromatography (GC-2014ATFSPL Shimadzu), detailed in previous works [36,37]. CO_2_ sorption tests were performed at a range of equilibrium pressure (1–30 bar) and temperatures of 25 °C, 45 °C and 65 °C. CO_2_/N_2_ selectivity was measured at 45 °C and equilibrium pressure of ∼27 bar. All tests were performed in triplicates. The stability was evaluated by ten CO_2_ sorption/desorption cycles at 45 °C and 4.3 bar with desorption following each cycle by heating at 70 °C for 1 h.

### 2.4 Statistical analysis

Minitab 18 Statistical Software-ANOVA was used to carry out statistical analysis to assess tests standard deviation (performed in triplicate) and analyze the Tukey test with 95% reliability. Equal letters show statistical equivalence of the sample averages. Also, temperature and pressure parameters were optimized by analyzing the surface and contour graph.

## 3 Results and discussion

**Fig. 2:**
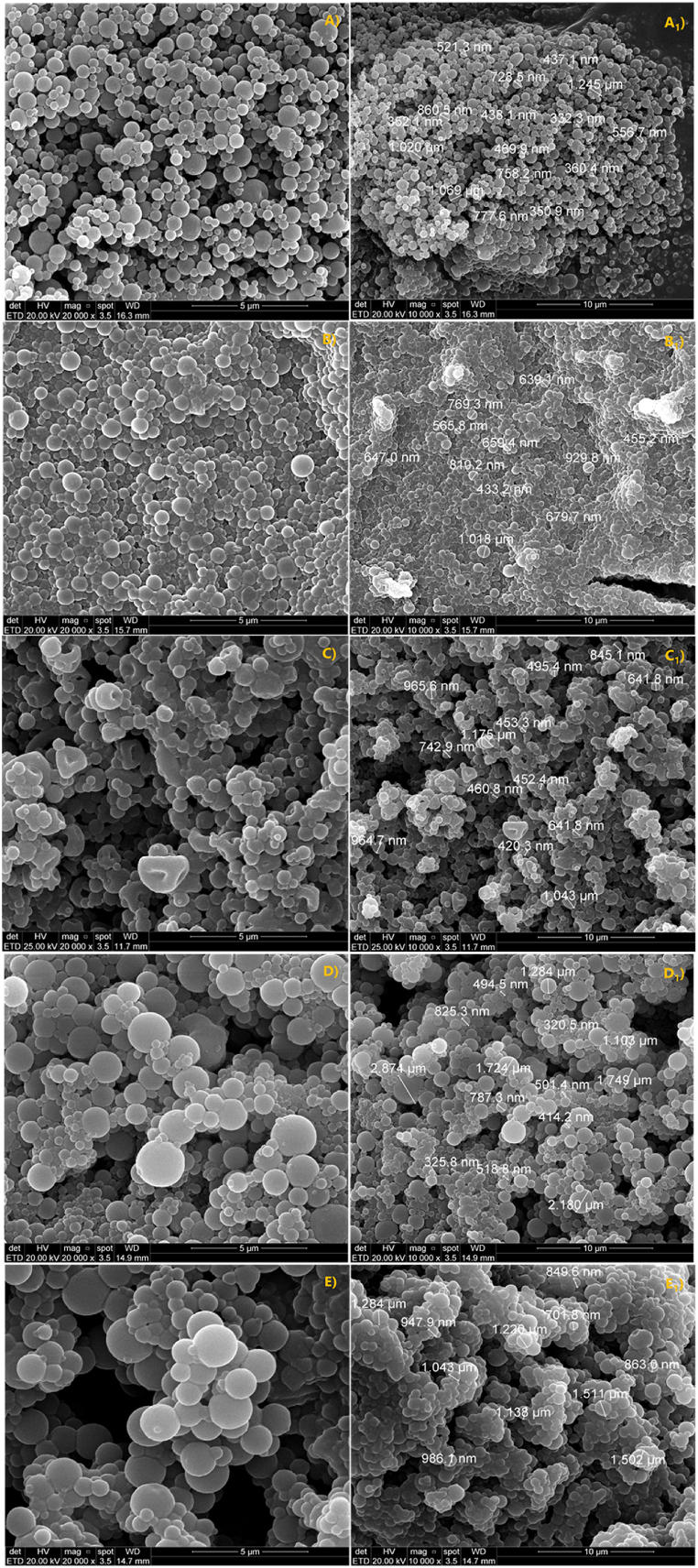
SEM, 20.000x magnification and SEM with particle size(_1_), 10.000x magnification: A) P[DADMA]-cap; B) P[DADMA]/MSO_3_; C) P[DADMA]/BF_4_; D) P[DADMA]/Br; E) P[DADMA]/CF_3_SO_3_.

Nanospray dryer is one of the technologies that allow obtaining nanoparticles [38]. Yet, by selecting an adequate polymer to obtain the shell one can produce nanoparticles using water as solvent. Fig. 2 shows SEM in two magnifications (20.000x - Fig. 2A–E and 10.000x - Fig. 2A_1_-E_1_) proving the success of using this kind of technology to obtain green nanoparticles by combining water-based PILs as shell and ILs as core. Particles with spherical morphology were observed both in P[DADMA]-cap (Fig. 2A) and capsules having ionic liquid as core (Fig. 2B–E). Average diameters demonstrated nanometric sizes (Fig. 2A_1_: ∼665 nm (+-266); Fig. 2B_1_: ∼695 nm (+-190); Fig. 2C_1_: ∼ ∼678 nm (+-222); Fig. 2D_1_: ∼718 nm (+-260); e Fig. 2E_1_: ∼931 nm (+-300)). EDX (Fig. S1) confirmed the presence of well-determined elements and uniform distribution of colors corresponding to C (red), F (green), S (blue), O (pink) and Br (yellow), confirming encapsulation and IL homogeneous distribution.

**Fig. 3:**
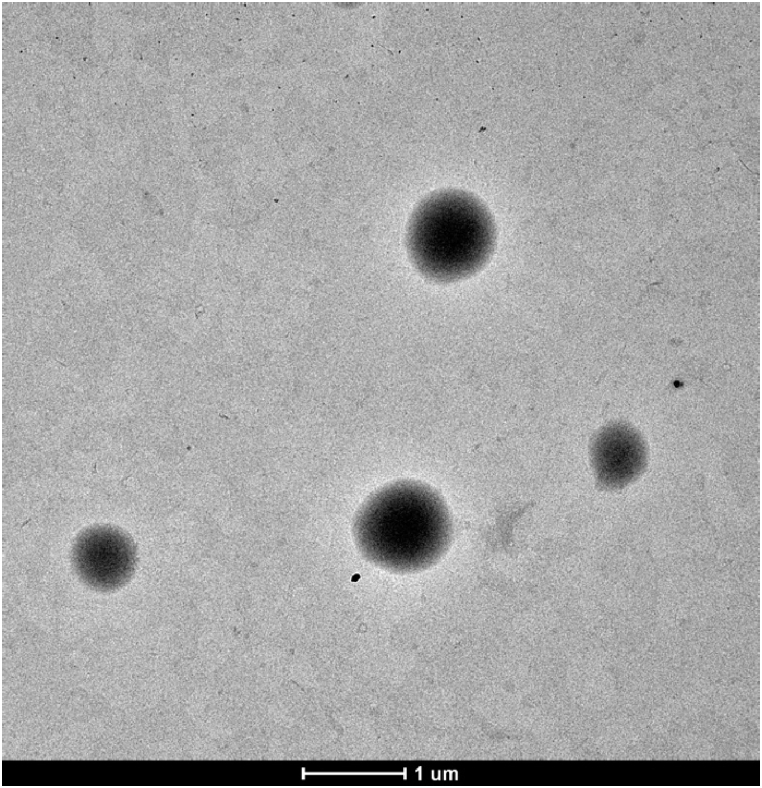
TEM P[DADMA]/MSO_3_.

In terms of shape and construction, particles can be called capsules or spheres. For capsules, the confined liquid is surrounded by a well-defined polymer line, while for spheres, the core and shell are mixed [39]. Capsules can be observed in TEM images (Fig. 3) and it represents the typical behavior of all particles obtained in this work.

FTIR also confirmed Emim[X] encapsulation within the P[DADMA][BF_4_] shell. All samples showed characteristic peaks of (see Fig. S2) shell material - cation poly(diallyldimethylammonium: 3058 cm^−1^ (C–H of N–CH_2_), 2946–2869 cm^−1^ (C–H of CH_3_), 1482–1387 cm^−1^ (C–H), 1286 cm^−1^ (N–C); and tetrafluoroborate anion: 1037-897 cm^−1^ (B–F) [30,40]. Characteristic peaks of ionic liquid were observed in encapsulated samples as cation [emim] [41]: 3170–3121 cm^−1^ (C–H aromatic), 1570 cm^−1^ (C=C aromatic), 1230 cm^−1^ (C–N aromatic), 1170 cm^−1^ (C–N aliphatic); and anions [42,43] [MSO_3_]: 757 cm^−1^ (C–S); [CF_3_SO_3_]: 1055 cm^−1^ (S=O), 754–631 cm^−1^ (C–F); [Br]: 617 cm^−1^. Other bands indicate O–H deformations, referring to water.

Encapsulation yield range is directly affected by the encapsulation technique [38]. IL encapsulated amount also can be influenced by the preparation method [23]. Wang et al. [23] compared IL loading amounts obtained by three different encapsulation methods (sol-gel, suspension polymerization and solvent evaporation) and observed that the first two presented higher values. Classical spray dryer shows a maximum yield of 70% [28], while for the nanospray dryer the encapsulation yield achieved 82.9% (seeTable 3).

**Fig. 4:**
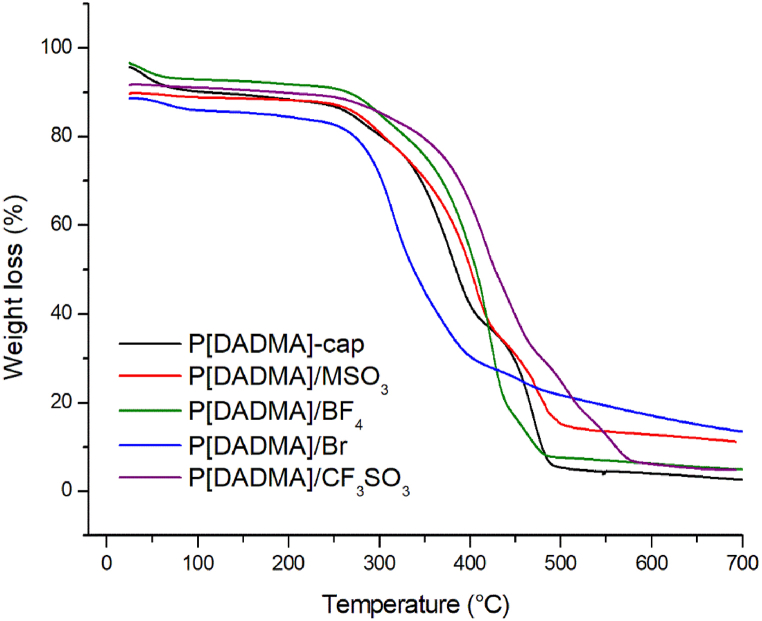
Capsules TGA analysis.

TGA curves are presented in Fig. 4 (see Table S1 for more information). Without considering the moisture loss at the beginning of the test, the P[DADMA]-cap exhibited other two main degradation stages [40]. First, the T_onset_ was observed at, approximately, 336.2 °C, attributed to [BF_4_]^-^ remotion and the loss of two methyl groups. The second T_onset2_, at 486.6 °C, refers to the complete polymer degradation [44]. With IL encapsulation, except for P[DADMA]/Br, thermal stability was improved, indicating that the anion plays an important role on determining thermal stability [45]. Founds of residual weights were in agreement with % IL encapsulated (Table 3 and Table S1) [20]. Differential scanning calorimetry (DSC) (see Table S2) indicated PLI amorphous structure, (T_g_ = −44.1 °C), presenting no endothermic peak [46]. Similar thermal transition profiles for encapsulated IL and pristine IL support the presence of the IL core [47]. P[DADMA]/Br showed no peaks in the test temperature, despite similar behavior reported in literature it requires further investigation to be fully understood [48].

**Table 3:** Process parameters: Yield (%), IL encapsulated amount (% IL) and encapsulation efficiency (EE).

Sample	Yield (%)	% IL	EE (%)
P[DADMA]-cap	74.4	–	–
P[DADMA]/MSO_3_	75.0	30.8 (+- 0.3)	69.2
P[DADMA]/BF_4_	72.0	31.4 (+- 0.4)	67.8
P[DADMA]/Br	82.9	27.0 (+- 1.4)	67.1
P[DADMA]/CF_3_SO_3_	77.9	32.1 (+- 0.4)	75.1

**Fig. 5:**
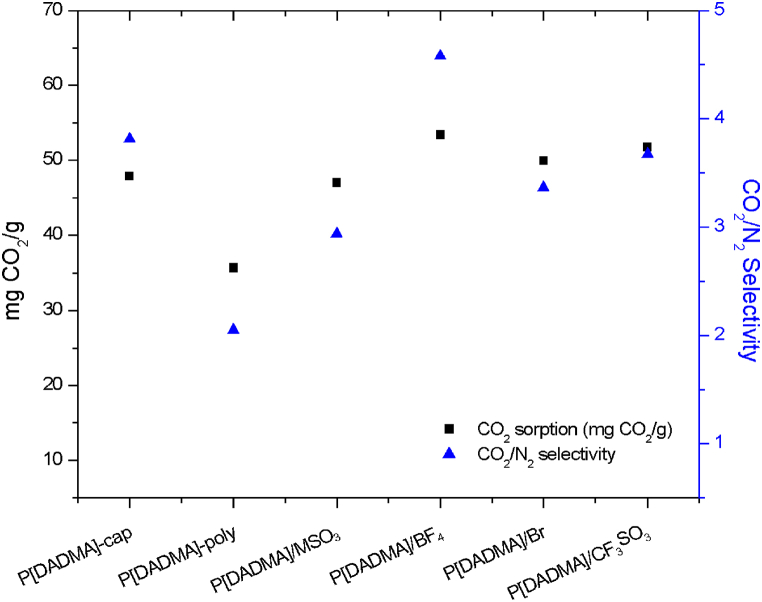
CO_2_ sorption (mg CO_2_/g) and CO_2_/N_2_ selectivity.

Fig. 5 demonstrates CO_2_ sorption (at ∼4.3 bar and 45 °C) and CO_2_/N_2_ selectivity results.

An increase in CO_2_ solubility (P[DADMA]-poly: 35.65 (±0.42) mg/g; P[DADMA]-cap: 47.88 (±1.55) mg/g) and CO_2_/N_2_ selectivity (P[DADMA]-poly: 2.05 (±0.23); P[DADMA]-cap: 3.81 (±0.14)) is noticed when comparing two different configuration of poly(ionic liquid) in capsule (P[DADMA]-cap) and solid polymer (P[DADMA]-poly), suggesting capsule formation can increase the active surface area, improving gas contact [17,18]. IL encapsulation showed even better results, indicating a synergic interaction between shell material and the ionic liquid. CO_2_ sorption best results were found for P[DADMA]/BF_4_ (53.40 (±0.39) mg/g) and P[DADMA]/CF_3_SO_3_ (51.76 (±0.26) mg/g), representing statistical equality (see Table S3). Ionic liquids with lower viscosity tend to favor CO_2_ permeability and diffusivity (μ (mPa.s): Emim[BF_4_] = 20.41 (40 °C) [49]; Emim[CF_3_SO_3_] = 23.86 (45 °C) [50]; Emim[MSO_3_] = 69 (45 °C) [51]; Emim[Br] = solid (45 °C) [52]), also fluorinated anions present high CO_2_ affinity [53,54]. These factors can be contributing for the obtained CO_2_ sorption results. Higher CO_2_/N_2_ selectivity was evidenced by P[DADMA]/BF_4_ (4.58 (±0.39)). ILs with smaller and symmetric anions tend to favor cavity creation and consequently a closer contact with CO_2_, improving CO_2_/N_2_ selectivity [[55], [56], [57]], corroborating IL Emim[BF_4_] best performance.

Emim[BF_4_] encapsulation with poly(diallyldimethylammonium tetrafluoroborate) as shell appears as a potential option for CO_2_ capture. The CO_2_ sorption capacities of samples obtained in this work are higher compared with some results reported in the literature, under similar conditions (Table 4).

**Table 4:** CO_2_ sorption (mg CO_2_/g) comparison with different encapsulated ILs.

Shell	Ionic Liquid	Conditions (T, P and %IL)	mg CO_2_/g	Ref.
PSF	Emim[Tf_2_N]	45 °C, 4 bar–37,5% (w)	44.2	[20]
PSF	Bmim[Tf_2_N]	45 °C, 4 bar–48%(w)	46.1	[19]
PVDF-HFP	Hmim[Tf_2_N]	23 °C, ∼4.5 bar 20% (w)	∼24.9	[17]
C_cap_	Bmim[GLY]	45 °C, 5 bar 55% (w)	∼50	[15]
C_cap_	Bmim[PRO]	45 °C, 5 bar 55% (w)	∼40	[15]
**P[DADMA]/[BF**_**4**_**]**	**Emim[BF**_**4**_**]**	**45°C, 4.3 bar ∼31.4 (w)**	**53.4**	**This Work**

PSF: Polysulfone; PVDF-HFP: Poly(vinylidene fluoride-*co*-hexafluoropropylene); C_cap_: hollow carbon.

CO_2_ sorption capacity of encapsulated ionic liquid tends to increase when compared with pristine ionic liquids. The encapsulation of Emim[BF_4_] (P[DADMA]/BF_4_) increased, approximately, four times the CO_2_ solubility when compared with the CO_2_ sorption capacity of the pristine IL Emim[BF_4_] reported in literature [58] (compare 53 mg/g CO_2_ at 45 °C and 4.3 bar to ∼13 mg/g CO_2_, at 40 °C and ∼5 bar). CO_2_ sorption kinetics can be drastically increased with encapsulation (see Fig. S3). Due to high viscosity, ILs take minutes or hours to achieve stability while encapsulated ionic liquid achieves this in seconds [17,20,37,59].

**Fig. 6:**
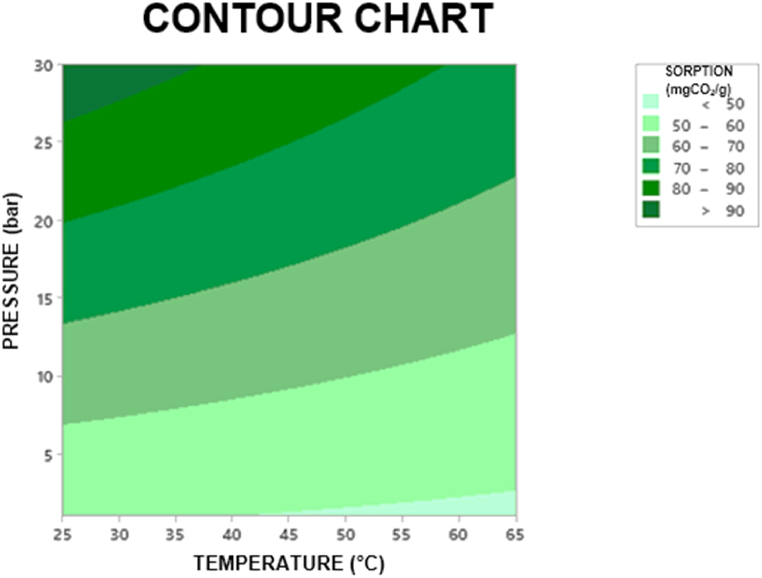
Contour chart for P[DADMA]/BF_4_ CO_2_ sorption at different temperatures and pressures.

Response surface analysis for P[DADMA]/BF_4_ was applied to determine the best conditions for CO_2_ capture (Table S5; Fig. S4). Results showed CO_2_ sorption capacity improvement with higher pressure and lower temperature (Fig. 6), typical behavior of physical absorption [60]. To achieve the highest CO_2_ sorption, a higher pressure (30 bar) and temperature of around 25 °C will be needed.

**Fig. 7:**
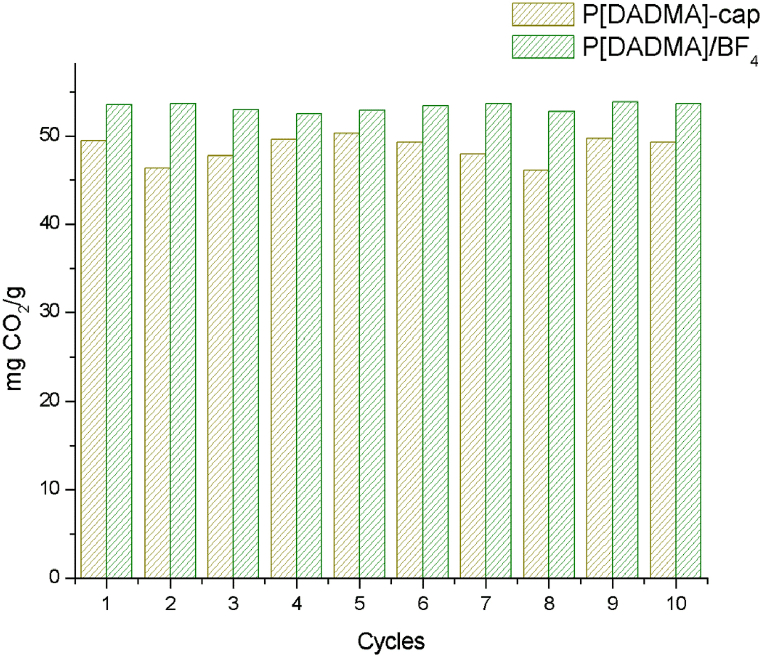
Recycle test at ∼4.3 bar and 45 °C.

Recycling tests are important in the development of new materials for CO_2_ capture. P[DADMA]-cap and P[DADMA]/BF_4_ were tested for ten cycles of CO_2_ sorption/desorption and from Fig. 7 we can see the CO_2_ soption capacity of both are still the same, keeping the deviation stability (Table S6), suggesting P[DADMA]-cap and P[DADMA]/BF_4_ were reversible for ten consecutive sorption/desorption cycles. Encapsulation of ionic liquids is suggested as an alternative to the leaching problem that occurs in some immobilization methods [13,16,18,21].

**Fig. 8:**
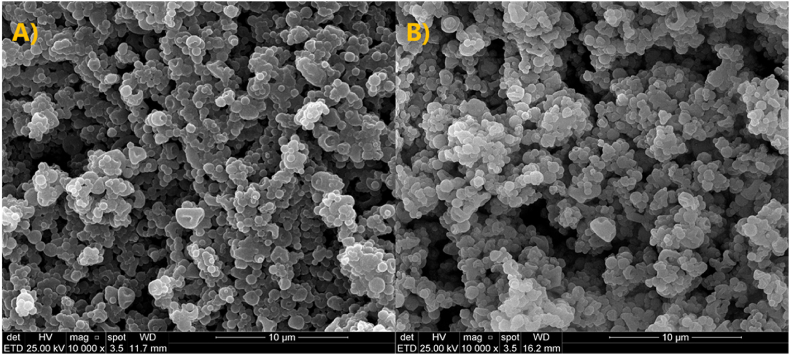
P[DADMA]/BF_4_ capsule stability. A) Before 10 cycles; B) After 10 cycles.

Aiming to confirm the stability of P[DADMA]/BF_4_ capsule after ten cycles, SEM image and acetone extraction test were performed. From Fig. 8 we can see that the particles shape keep the same before (Fig. 8A) and after ten cycles (Fig. 8B). Capsules good stability was confirmed for both techniques as seen in Fig. 8 by the maintenance of capsules configuration and the encapsulated IL % content (30.9%).

## 4 Conclusions

Capsules of water-based poly(ionic liquid) P[DADMA][BF_4_] and encapsulated ionic liquids Emim[X] were obtained for the first time using Nano Spray Dryer B-90. The combination of these promising materials for CO_2_ capture promoted gains for CO_2_ sorption, CO_2_/N_2_ selectivity, thermal stability and CO_2_ sorption kinetic proving to be stable under use, emphasizing this as a potential alternative. P[DADMA]/BF_4_ showed the best results for CO_2_ capture. It must be emphasized that this new material for CO_2_ capture is organic solvent-free in the encapsulation step helping also to create new options for the development of green processes.

## Author contribution statement

Bárbara B. Polesso: Conceived and designed the experiments; Performed the experiments; Analyzed and interpreted the data; Contributed reagents, materials, analysis tools or data; Wrote the paper.

Rafael Duczinski: Performed the experiments; Analyzed and interpreted the data; Wrote the paper.

Franciele L. Bernard: Conceived and designed the experiments; Analyzed and interpreted the data; Wrote the paper.

Douglas J. Faria: Analyzed and interpreted the data; Wrote the paper.

Leonardo M. dos Santos: Performed the experiments; Analyzed and interpreted the data; Contributed reagents, materials, analysis tools or data; Wrote the paper.

Sandra Einloft: Conceived and designed the experiments; Analyzed and interpreted the data; Contributed reagents, materials, analysis tools or data; Wrote the paper.

## Funding statement

Rafael Duczinski was supported by Shell Brasil [4610056697]. Bárbara B. Polesso was supported by capes [001]. Professor Sandra Einloft was supported by CNPq [316580/2021-0]. This work was supported by fapergs [21/2551-0002235-3].

## Data availability statement

Data included in article/supp. material/referenced in article.

## Declaration of interest’s statement

The authors declare no competing interests.
