# Supplementary material for: New water-based nanocapsules of poly(diallyldimethylammonium tetrafluoroborate)/ionic liquid for CO2 capture
Source: Heliyon. 2023 Jan 30;9(2):e13298. doi: 10.1016/j.heliyon.2023.e13298 (PMC9900371; doi:10.1016/j.heliyon.2023.e13298)
Supplement: Multimedia component 1 [file mmc1.docx]

**[Supplementary information](http://www.nature.com/nmat/journal/v8/n7/suppinfo/nmat2469_S1.html)**

New water-based nanocapsules of poly(diallyldimethylammonium tetrafluoroborate)/ionic liquid for CO_2_ capture

Bárbara B. Polesso^1^, Rafael Duczinski^1^, Franciele L. Bernard^2^, Douglas J. Faria^1^, Leonardo M. dos Santos^2^, Sandra Einloft^1,2*^

^1^ Post-Graduation Program in Materials Engineering and Technology, Pontifical Catholic University of Rio Grande do Sul – PUCRS, Brazil;

^2^ School of Technology, Pontifical Catholic University of Rio Grande do Sul – PUCRS, Brazil;

# **Corresponding author:* [*einloft@pucrs.br*](mailto:einloft@pucrs.br)

Fig.S1. SEM-EDX(_1_): A) P[DADMA]-cap; B) P[DADMA]/MSO_3_; C) P[DADMA]/BF_4_; D) P[DADMA]/Br; E) P[DADMA]/CF_3_SO_3_





Fig.S2. FTIR capsules.





Fig.S3. CO_2_ sorption time of P[DADMA][BF_4_] .


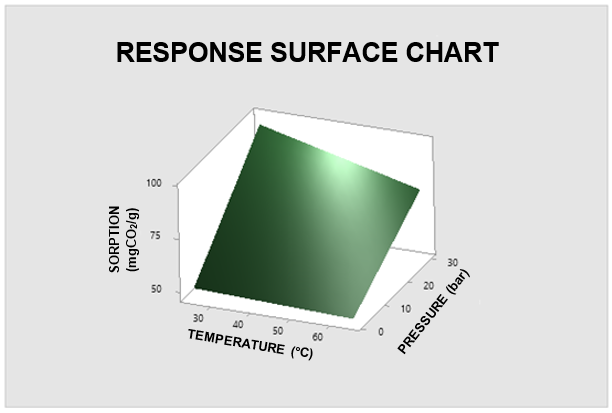


Fig.S4. Response Surface Chart of P[DADMA][BF_4_] .

Table S1. TGA

| Sample | T_onset1_  (°C) | T_onset2_  (°C) | Residue  % |
| --- | --- | --- | --- |
| P[DADMA]-cap | 336.2 | 486.6 | 2.60 |
| P[DADMA]/MSO_3_ | 347.7 | 491.6 | 11.22 |
| P[DADMA]/BF_4_ | 380.0 | 488.5 | 4.94 |
| P[DADMA]/Br | 288.2 | 509.2 | 13.43 |
| P[DADMA]/CF_3_SO_3_ | 366.8 | 558.2 | 4.90 |

Table S2. DSC

| Sample | T_g_  (°C) | T_c_  (°C) | T_m_  (°C) |
| --- | --- | --- | --- |
| P[DADMA]-cap | -44.1 | - | - |
| P[DADMA]/MSO_3_ | - | - | - |
| P[DADMA]/BF_4_ | - | - | -56.3 |
| P[DADMA]/Br | -51.2 | - | - |
| P[DADMA]/CF_3_SO_3_ | - | -75.6 | -59.9 |
| Emim[MSO_3_] | - | - | - |
| Emim[BF_4_] | - | - | -40.0 |
| Emim[Br] | - | - | 82.1 |
| Emim[CF_3_SO_3_] | - | -69.3 | -46.0 |

Table S3. Tukey test CO_2_ sorption (mg CO_2_/g)

Sample N Average Tukey Test

P[DADMA]/BF_4_ 3 53.4033 A

P[DADMA]/CF_3_SO_3_ 3 51.7633 A B

P[DADMA]/Br 3 49.9733 B C

P[DADMA] - cap 3 47.8833 C D

P[DADMA]/MSO_3_ 3 46.9933 D

P[DADMA] - poly 3 35.6533 E

Table S4. Tukey test CO_2_/N_2_ Selectivity

Sample N Average Tukey Test

P[DADMA]/BF_4_ 3 4.58000 A

P[DADMA] - cap 3 3.81333 B

P[DADMA]/CF_3_SO_3_ 3 3.67333 B

P[DADMA]/Br 3 3.36333 B C

P[DADMA]/MSO_3_ 3 2.93333 C

P[DADMA] - poly 3 2.05000 D

Table S5. Response surface methodology using Minitab

| StdOrder | RunOrder | PtType | Blocks | T (°C) | P (bar) | mg CO_2_/g |
| --- | --- | --- | --- | --- | --- | --- |
| 44 | 1 | 1 | 1 | 25 | 4 | 54.54 |
| 31 | 2 | 1 | 1 | 45 | 10 | 63.63 |
| 17 | 3 | 1 | 1 | 65 | 10 | 62.36 |
| 52 | 4 | 1 | 1 | 45 | 10 | 62.63 |
| 7 | 5 | 1 | 1 | 25 | 30 | 108.6 |
| 45 | 6 | 1 | 1 | 25 | 10 | 64.87 |
| 4 | 7 | 1 | 1 | 25 | 15 | 78.63 |
| 29 | 8 | 1 | 1 | 45 | 1 | 43.74 |
| 14 | 9 | 1 | 1 | 45 | 30 | 81.54 |
| 54 | 10 | 1 | 1 | 45 | 20 | 75.89 |
| 40 | 11 | 1 | 1 | 65 | 20 | 68.43 |
| 16 | 12 | 1 | 1 | 65 | 4 | 50.64 |
| 63 | 13 | 1 | 1 | 65 | 30 | 75.67 |
| 18 | 14 | 1 | 1 | 65 | 15 | 65.4 |
| 55 | 15 | 1 | 1 | 45 | 25 | 79.96 |
| 19 | 16 | 1 | 1 | 65 | 20 | 69.3 |
| 27 | 17 | 1 | 1 | 25 | 25 | 106.73 |
| 23 | 18 | 1 | 1 | 25 | 4 | 54.11 |
| 34 | 19 | 1 | 1 | 45 | 25 | 78.83 |
| 12 | 20 | 1 | 1 | 45 | 20 | 74.98 |
| 8 | 21 | 1 | 1 | 45 | 1 | 44.77 |
| 62 | 22 | 1 | 1 | 65 | 25 | 71.26 |
| 9 | 23 | 1 | 1 | 45 | 4 | 52.95 |
| 56 | 24 | 1 | 1 | 45 | 30 | 81.88 |
| 21 | 25 | 1 | 1 | 65 | 30 | 75.2 |
| 57 | 26 | 1 | 1 | 65 | 1 | 42.76 |
| 47 | 27 | 1 | 1 | 25 | 20 | 83.95 |
| 10 | 28 | 1 | 1 | 45 | 10 | 64.13 |
| 32 | 29 | 1 | 1 | 45 | 15 | 70.25 |
| 41 | 30 | 1 | 1 | 65 | 25 | 71.95 |
| 58 | 31 | 1 | 1 | 65 | 4 | 50.63 |
| 37 | 32 | 1 | 1 | 65 | 4 | 51.44 |
| 15 | 33 | 1 | 1 | 65 | 1 | 44 |
| 53 | 34 | 1 | 1 | 45 | 15 | 70.59 |
| 11 | 35 | 1 | 1 | 45 | 15 | 71.72 |
| 49 | 36 | 1 | 1 | 25 | 30 | 109.95 |
| 3 | 37 | 1 | 1 | 25 | 10 | 65.85 |
| 48 | 38 | 1 | 1 | 25 | 25 | 105.84 |
| 13 | 39 | 1 | 1 | 45 | 25 | 78.99 |
| 6 | 40 | 1 | 1 | 25 | 25 | 105.27 |
| 30 | 41 | 1 | 1 | 45 | 4 | 53.7 |
| 20 | 42 | 1 | 1 | 65 | 25 | 71.77 |
| 43 | 43 | 1 | 1 | 25 | 1 | 45.58 |
| 22 | 44 | 1 | 1 | 25 | 1 | 45.15 |
| 25 | 45 | 1 | 1 | 25 | 15 | 78.12 |
| 42 | 46 | 1 | 1 | 65 | 30 | 72.94 |
| 1 | 47 | 1 | 1 | 25 | 1 | 43.74 |
| 46 | 48 | 1 | 1 | 25 | 15 | 77,48 |
| 36 | 49 | 1 | 1 | 65 | 1 | 44.8 |
| 35 | 50 | 1 | 1 | 45 | 30 | 81.34 |
| 51 | 51 | 1 | 1 | 45 | 4 | 53.56 |
| 33 | 52 | 1 | 1 | 45 | 20 | 75.89 |
| 59 | 53 | 1 | 1 | 65 | 10 | 62.27 |
| 5 | 54 | 1 | 1 | 25 | 20 | 84.99 |
| 28 | 55 | 1 | 1 | 25 | 30 | 11,21 |
| 2 | 56 | 1 | 1 | 25 | 4 | 54.54 |
| 60 | 57 | 1 | 1 | 65 | 15 | 66.83 |
| 38 | 58 | 1 | 1 | 65 | 10 | 62.17 |
| 24 | 59 | 1 | 1 | 25 | 10 | 66.25 |
| 50 | 60 | 1 | 1 | 45 | 1 | 43.97 |
| 39 | 61 | 1 | 1 | 65 | 15 | 65.94 |
| 26 | 62 | 1 | 1 | 25 | 20 | 85.95 |
| 61 | 63 | 1 | 1 | 65 | 20 | 68.51 |

Table S6. CO_2_ sorption (mg CO_2_/g) recycle test

| Cycle | P[DADMA]-cap | P[DADMA]/BF_4_ |
| --- | --- | --- |
| 1 | 49.50 | 53.56 |
| 2 | 46.40 | 53.70 |
| 3 | 47.75 | 52.95 |
| 4 | 49.61 | 52.52 |
| 5 | 50.31 | 52.90 |
| 6 | 49.32 | 53.41 |
| 7 | 47.94 | 53.69 |
| 8 | 46.13 | 52.76 |
| 9 | 49.74 | 53.89 |
| 10 | 49.32 | 53.65 |
